# Co-culturing a novel *Bacillus* strain with *Clostridium tyrobutyricum* ATCC 25755 to produce butyric acid from sucrose

**DOI:** 10.1186/1754-6834-6-35

**Published:** 2013-03-04

**Authors:** Mohammed Dwidar, Seil Kim, Byoung Seung Jeon, Youngsoon Um, Robert J Mitchell, Byoung-In Sang

**Affiliations:** 1 Clean Energy Center, Korea Institute of Science and Technology, Seoul, 136-791, Republic of Korea; 2 School of Nano-Bioscience and Chemical Engineering, Ulsan National Institute of Science and Technology, Ulsan, 689-798, South Korea; 3 Department of Applied Chemical Engineering, Hanyang University, Seoul, Republic of Korea

**Keywords:** Butyric acid, Sucrose, Levansucrase, Co-culture, *Clostridium tyrobutyricum* ATCC 25755, *Bacillu*s sp. SGP1

## Abstract

**Background:**

Currently, the most promising microorganism used for the bio-production of butyric acid is *Clostridium tyrobutyricum* ATCC 25755^T^; however, it is unable to use sucrose as a sole carbon source. Consequently, a newly isolated strain, *Bacillu*s sp. SGP1, that was found to produce a levansucrase enzyme, which hydrolyzes sucrose into fructose and glucose, was used in a co-culture with this strain, permitting *C. tyrobutyricum* ATCC 25755^T^ to ferment sucrose to butyric acid.

**Results:**

*B.* sp. SGP1 alone did not show any butyric acid production and the main metabolite produced was lactic acid. This allowed *C. tyrobutyricum* ATCC 25755^T^ to utilize the monosaccharides resulting from the activity of levansucrase together with the lactic acid produced by *B.* sp. SGP1 to generate butyric acid, which was the main fermentative product within the co-culture. Furthermore, the final acetic acid concentration in the co-culture was significantly lower when compared with pure *C. tyrobutyricum* ATCC 25755^T^ cultures grown on glucose. In fed-batch fermentations, the optimum conditions for the production of butyric acid were around pH 5.50 and a temperature of 37°C. Under these conditions, the final butyrate concentration was 34.2±1.8 g/L with yields of 0.35±0.03 g _butyrate_/g _sucrose_ and maximum productivity of 0.3±0.04 g/L/h.

**Conclusions:**

Using this co-culture, sucrose can be utilized as a carbon source for butyric acid production at a relatively high yield. In addition, this co-culture offers also the benefit of a greater selectivity, with butyric acid constituting 92.8% of the acids when the fermentation was terminated.

## Background

Butyric acid has several potential applications in chemical and pharmaceutical industries and can be useful for fuel production [1]. It is also widely used in foodstuffs and beverage industries [2]. Nowadays, butyric acid is produced mainly for industry from crude oil through petrochemical methods [2]. However, butyric acid obtained from microbial fermentation is more promising due to the growing needs for bio-based natural products especially for food additives, and pharmaceuticals [3].

Currently, the most promising microorganism used for the bio-production of butyric acid is *Clostridium tyrobutyricum* ATCC 25755^T^[4-7]. This strain is capable of producing butyric acid with high selectivity and can tolerate high concentrations. However, it can only ferment monosaccharides like glucose, xylose and fructose, and is currently unable to utilize disaccharides, such as sucrose and lactose [8-10]. Despite the presence of several butyric acid bacteria which can utilize sucrose as a carbon source, such as *Clostridium butyricum*[11,12] and some strains of *C. tyrobutyricum*, such as *C. tyrobutyricum* ZJU8235 [13], *C. tyrobutyricum* ATCC 25755^T^ offers the highest butyric acid yields and final concentrations and so is the preferred strain.

Since the cost of culture media components has a great influence on the overall cost of butyric acid produced by fermentation [2,10], we wanted to utilize cheaper substrates for butyric acid production. One such substrate, sucrose, is an important and relatively cheap carbon source and is readily available in nature. It forms more than 90% of the total carbohydrates in sugar canes [14] and sugar beet in which sugars can account for 12% to 20% of the plant's dry weight. Sucrose also constitutes large percentage of the total sugar content in molasses. Consequently, our lab seeks to use sucrose as a carbon source for butyric acid production.

In previous studies, several research groups evaluated the use of co-culturing to address limitation in substrate utilization by individual strains for the eventual production of different fermentation products. For example, Zhang et al. (2009) [3] discussed the possibility of using *Clostridium thermocellum* in a co-culture with *Clostridium thermobutyricum* for butyric acid production from cellulose, while Chang et al. (2008) [15] reported that the combination of aerobic *Bacillus* and anaerobic *Clostridium* may play the key role in the future of biofuel production from biomass. Their rationale was that strains of *Bacillus* generally have a high growth rate and secrete many saccharification-related extracellular enzymes into the medium, such as amylase, pectinase, protease, cellulose, and hemicellulase. Trana et al. (2010) [16] have recently shown the potential of co-culturing *Bacillus subtilis* with *Clostridium butylicum* for butanol production from starch. However, the *Bacillus* species used in the above studies are aerobic and, as such, they cannot be stably used in co-cultures for long periods with Clostridia, which are anaerobic. In contrast, *Bacillus* strains which are facultative anaerobes such as *Bacillus licheniformis*, will be more appropriate for long term co-culturing with *Clostridium* strains under anaerobic conditions.

In this study, we characterized the levansucrase activity of a newly isolated strain of *Bacillus* which was found to be closely related to *B.licheniformis* ATCC 14580^T^ and *Bacillus sonorensis* NRRL B-23154^T^ based upon 16S rRNA gene sequencing. To demonstrate its applicability, we next performed co-culture fermentations with this strain and *C. tyrobutyricum* ATCC 25755^T^ for butyric acid production using sucrose as a carbon source.

## Results and discussion

### *B.* sp. SGP1 Hydrolyzes sucrose through levansucrase

*B.* sp. SGP1 was isolated from a waste water sludge sample obtained from anaerobic digestion tank. Identifying this strain through 16S rRNA gene sequencing revealed that its closest relative was *Bacillus licheniformis* ATCC 14580^T^ (99.4%, 16S rRNA gene similarity) followed by *Bacillus sonorensis* NRRL B-23154^T^ (99.3%) and *Bacillus aerius* 24K^T^ (99.3%). The phylogenetic analysis (Additional file 1: Figure S1) showed that the strain SGP1 formed a monophyletic group with *B. licheniformis* ATCC 14580^T^, *B. sonorensis* NRRL B-23154^T^ and *B. aerius* 24K^T^.

**Figure 1:**
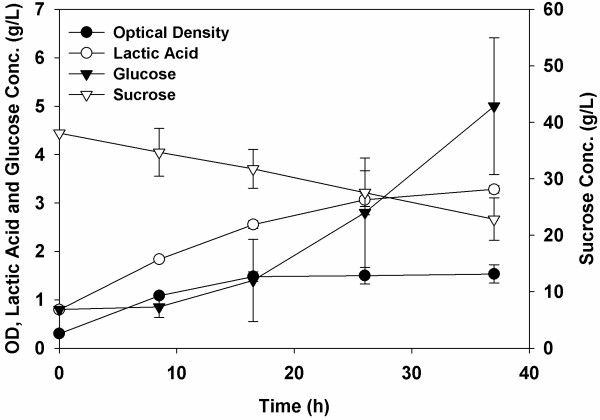
**Growth profile of *B. *sp. SGP1 when cultured on RCM supplemented with sucrose in serum bottle at 37°C under anoxic conditions.**

When *B.* sp. SGP1 was cultured alone on sucrose in batch cultures, lactic acid was the main fermentation product (Figure 1). After 16 h of growth, the optical density leveled off and remained steady for about 20 h longer. During this time, though, sucrose hydrolysis continued as evidenced by the increasing concentration of glucose found within the culture supernatant.

Many groups reported previously that several *Bacillus* strains, and specifically *B. licheniformis*, are able to produce levansucrase. This enzyme can both hydrolyze sucrose into its constituent glucose and fructose subunits and form the levan polymer from the fructose subunits [17,18]. Previous studies demonstrated that levansucrase also degrades levan to individual fructose monomers especially when the reducing sugar concentration is low [18-20]. Consequently, we tested for the production of levansucrase by *B.* sp. SGP1. For colonies grown on agar plates containing sucrose, the production of a polysaccharide was obvious as they showed a slimy mucoid growth. In contrast, when grown on agar plates containing glucose, none of the colonies were mucoid. This polysaccharide was consequently precipitated from liquid cultures of *B.* sp. SGP1 grown on sucrose and hydrolyzed to its constituent monosaccharides. Analysis of these sugars by HPLC (High-performance liquid chromatography) found fructose monomers, implying that the polysaccharide was levan and that the extracellular enzyme is levansucrase.

**Figure 2:**
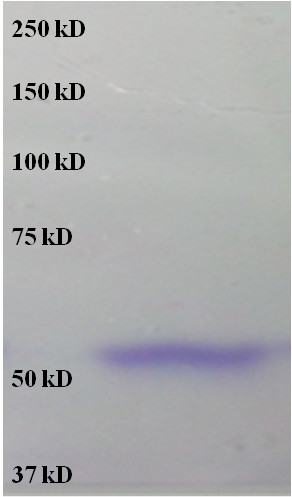
**Levansucrase molecular mass analysis by SDS-PAGE gel.** The band was revealed by *in situ* levan synthesis as described in the materials and methods.

**Figure 3:**
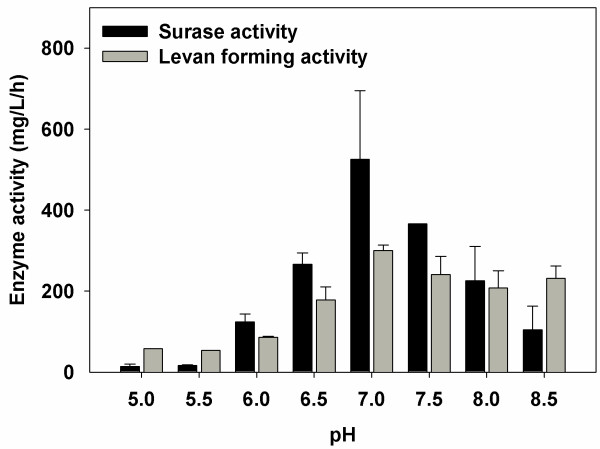
**Sucrase and levan forming activities of *B. *sp. SGP1 supernatant at different pH values.** The liberated glucose and levan concentrations were determined as described in the materials and methods.

To further confirm that the enzyme responsible is levansucrase, the proteins present in the supernatant of a 24 h old culture were precipitated and run on an SDS-page gel. After renaturing the proteins, the levan was formed and stained as described in the Materials and Methods. The presence of the levan polymer within the gel is clearly seen in Figure 2. Only one band was observed and it corresponded to a protein having a molecular weight of approximately 55 KDa, a weight that is similar to that of levansucrase enzymes from other species [20]. Further characterization established that this enzyme was optimally active at a neutral pH (Figure 3) and at temperatures of up to 43ºC, the highest temperature tested in this study (Additional file 2: Figure S2). Consequently, it can be used within co-cultures with many fermentative clostridia, which grow best at mesophilic temperatures.

### Co-culturing *B.* sp. SGP1 with *C. tyrobutyricum* ATCC 25755^T^

**Figure 4:**
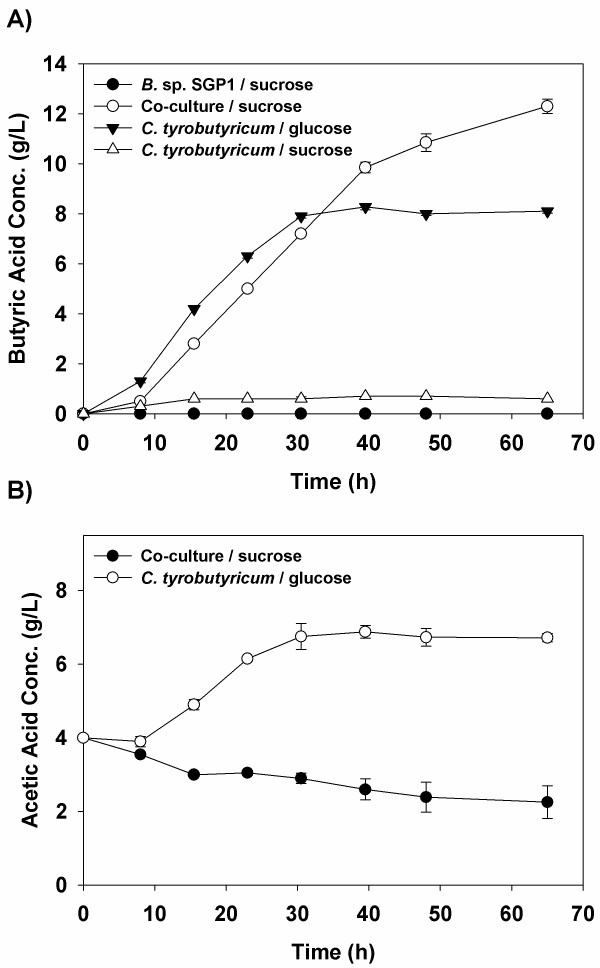
**A) Butyric acid production by individual and mixed cultures of *B. *sp. SGP1 and *C. tyrobutyricum *ATCC 25755**^**T**^**.** This experiment was performed using RCM supplemented with sucrose or glucose in serum bottles at 37°C under anoxic conditions. **B**) Plot showing the acetic acid concentration when *C. tyrobutyricum* ATCC 25755^T^ was cultured alone on glucose and from the co-culture grown on sucrose.

Since *B.* sp. SGP1 produces glucose during the hydrolysis of sucrose but does not continue to utilize or ferment it after its initial growth (Figure 1), we hypothesized that this strain can be used in conjunction with other fermentative strains and particularly those that show substrate limitations towards sucrose, such as *C. tyrobutyricum* ATCC 25755^T^[10]. To evaluate this, several cultures were grown using these two strains independently or together with either glucose or sucrose as the fermentative substrate. The cultures were evaluated based upon the amount of butyric acid produced over time (Figure 4). Growth of *C. tyrobutyricum* ATCC 25755^T^ or *B.* sp. SGP1 by themselves on RCM (Reinforced Clostridial Media) supplemented with sucrose gave little or no butyric acid, respectively. However, when these two strains were co-cultured on sucrose, a significant amount of butyric acid was produced, *i.e.*, 13.5 g/L (Figure 4A), indicating that *C. tyrobutyricum* ATCC 25755^T^ was able to ferment the monosaccharides produced by the levansucrase.

Interestingly, it was found that the amount of butyric acid produced by the co-culture in serum bottles using sucrose is actually higher than that produced from *C. tyrobutyricum* ATCC 25755^T^ when grown alone on the same amount of glucose (Figure 4A). In contrast, the final acetate concentration in the co-culture was significantly lower than that seen from *C. tyrobutyricum* ATCC 25755^T^ cultured solely on glucose (Figure 4B). These two findings are likely related as the final total acid concentrations within the cultures were similar, 14.6 g/L for the co-culture and 14.8 g/L for *C. tyrobutyricum*, indicating that the co-culture is capable of utilizing more of the acetate within the media and producing a subsequently higher amount of butyric acid. One possible explanation that can account for the lower acetate levels found in the co-culture is the glucose concentration, which was undetectable (< 0.1 g/L) during the exponential growth phase. Consequently, it would appear that it was consumed immediately by the culture as soon as it was produced from sucrose. Under similar glucose-limited conditions, other groups have found that *C. tyrobutyricum* ATCC 25755^T^ shifts more carbon to the butyrate pathway instead of acetate production [3,21].

**Figure 5:**
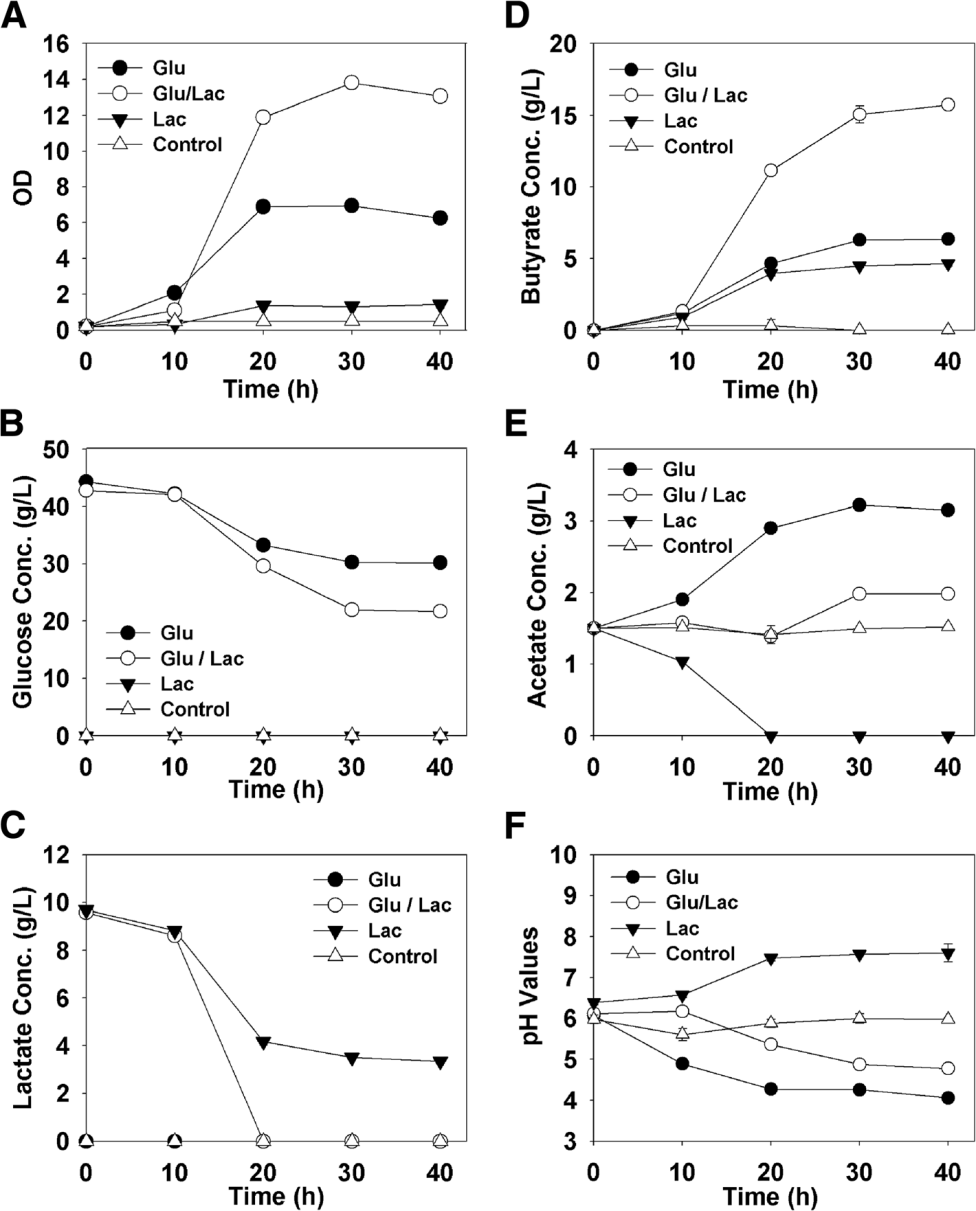
**Characteristics of *C. tyrobutyricum *ATCC 25755**^**T **^**cultures grown on P2 medium supplemented with either 40.0 g/L glucose (Glu), 40.0 g/L glucose and 10.0 g/L lactate (Glu+Lac), 10.0 g/L lactate (Lac) or nothing (Control).** This results shown are (**A**) optical density, (**B**) glucose concentration, (**C**) lactic acid concentration, (**D**) butyric acid concentration, (**E**) acetic acid concentration and (**F**) pH of the cultures.

Another possible reason for this is the activity of *B.* sp. SGP1, which produces lactic acid (Figure 1). Given that lactic acid was not detected within the co-culture, it appears that it and acetic acid are co-metabolized by *C. tyrobutyricum* ATCC 25755^T^ as fermentative substrates to produce butyric acid [8,9]. As evidence of this, batch cultures of *C. tyrobutyricum* ATCC 25755^T^ were grown using P2 medium supplemented with either glucose (40.0 g/L), lactic acid (10.0 g/L in the form of sodium lactate) or both (Figure 5). When lactic acid was added alone, both it and the acetate present in the media were consumed (Figure 5C and E), leading to butyric acid production (Figure 5D). The consumption of two acids and subsequent production of only one led to a significant increase in the pH as the molar concentration of acids was nearly halved, *i.e*., 0.025 M of acetate and 0.070 M lactate produced 0.053 M butyrate (Figure 5F). These results confirm that acetate and lactate can be co-utilized by *C. tyrobutyricum* ATCC 25755^T^ to produce butyric acid, with a yield under these conditions of 0.6 g butyrate/g (acetate and lactate). In fact, this is in agreement with the equation reported previously for acetate-lactate fermentation by *C. butyricum*, where:

$$\text{Acetate}+3\text{Lactate}+\text{ADP}+P_{i}\to 2\text{Butyrate}+{2H}_{2}+\text{ATP}$$

[22].

When lactic acid was added along with glucose, *C. tyrobutyricum* ATCC 25755^T^ showed an enhanced growth and butyric acid production, which can be in part attributed to the pH buffering effect described above. In confirmation of Figure 4A, the acetate concentration did not increase, as was seen when glucose was added alone, while the lactic acid concentration decreased, suggesting that these two acids were once more being converted into butyric acid.

### Lactic acid is converted only into butyric acid not acetic acid

**Figure 6:**
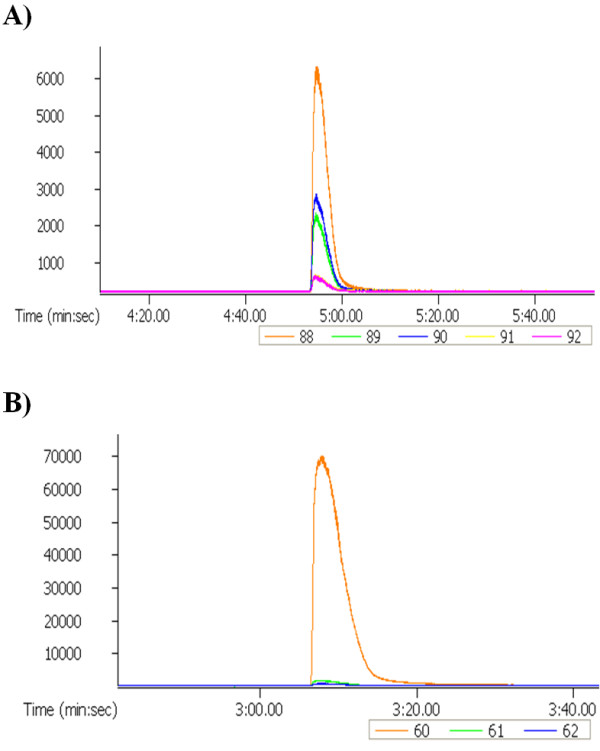
**GC-MS spectra of acetic and butyric acids present in the culture broth of *C. tyrobutyricum *ATCC 25755**^**T **^**after culturing for 40 h in P2 medium with **^**12**^**C**_**6**_**-glucose (40.0 g/L) and [1,2,3-**^**13**^**C**_**3**_**] lactic acid (5.0 g/L). A**) Mass spectra of butyric acid showing the presence of significant amounts of butyric acid with molecular weights higher than 88 (i.e.: 89, 90, 91 and 92) indicating the corporation of ^13^C. **B**) Mass spectra of acetic acid showing the absence of ^13^C in the acetic acid.

To confirm that lactic acid is being converted by *C. tyrobutyricum* ATCC 25755^T^ into butyric acid only, P2 medium was prepared once more but with a mixture of ^12^C- glucose (40.0 g/L) and [1, 2, 3 ^13^C_3_] lactic acid (5.0 g/L). After 40 h of culturing, samples were taken, and the resulting butyric and acetic acids were analyzed for the presence of ^13^C (Figure 6). Acetic acid showed only a single peak at 60 (m/z) corresponding to the expected molecular weight of ^12^C_2_-acetic acid. For butyric acid, however, we found multiple molecular peaks at 88 (m/z), which corresponds to the molecular weight of ^12^C_4_-butyric acid, as well as at 89, 90, 91 and 92 (m/z), indicating the existence of significant amounts of ^13^C in butyric acid. These results demonstrate that the lactic acid present in the media is being used as a substrate by *C. tyrobutyricum* ATCC 25755^T^ and that it is being converted only into butyric acid and not for acetic acid production even in presence of another carbon source in the medium such as glucose.

### Optimizing pH conditions in the co-culture for butyric acid production

The optimum pH for culturing *B.* sp. SGP1 was around 7.0 while more acidic conditions led to a decreased growth (Additional file 3: Figure S3). However, for *C. tyrobutyricum* ATCC 25755^T^, the optimum pH for growth and butyric acid production is known to be 5.9 to 6.0 [4-6,23], a value that we used previously to produce more than 50 g/L butyric acid [7]. For this co-culture, we tried fed-batch fermentations at different pH values (5.9, 5.7, 5.5, and 5.3). All fermentations were performed at 37°C as this temperature was found to be the best for the co-culture based upon butyric acid final concentration (Additional file 4: Figure S4).

**Figure 7:**
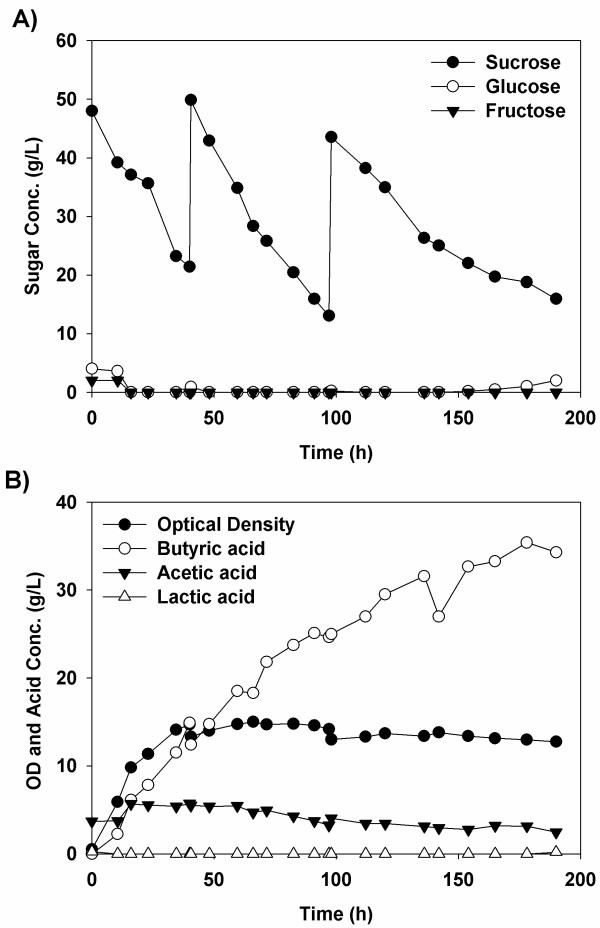
**Typical *B. *sp. SGP1 and *C. tyrobutyricum *ATCC 25755**^**T **^**co-culture fed-batch fermentation in RCM supplemented with sucrose at pH 5.5 and 37°C.** The plots show (**A**) the sugars consumption profile and (**B**)the optical density of the cultures and their production profiles for butyric, acetic and lactic acids.

Interestingly, the optimum pH for the co-culture both in terms of growth and butyric acid production was 5.5 (Figure 7) which is lower than the optimum for growth of either strain. At this pH, the maximum productivity was 0.3±0.04 g/L/h and the final butyrate concentration was 34.2±1.8 g/L with a yield of 0.35±0.03 g butyrate/g sucrose based upon the amount of sucrose consumed. By comparison, in a previous study done by our group employing immobilized *C. tyrobutyricum* ATCC 25755^T^, a yield of 0.45 g butyrate/g glucose was reported using the same media as in this study [7]. Another study looking at the fed-batch addition of carbonated beverages, which contain sucrose, glucose and fructose, to cultures of *C. tyrobutyricum* ATCC 25755^T^ grown in RCM media found yields of 0.42 ± 0.11 g/g [10], a value that is comparable with that seen in our study. The final acetate and lactate concentrations in the medium were 2.5 g/L and 0.2 g/L, respectively. Accordingly, this co-culture was selective for butyric acid, with 92.8% of the final acids by weight being butyric acid. By comparison, our previous study found butyric acid constituted only 82% of the total acids when 35.4 g/L butyric acid was produced during a fed batch culture with glucose [7].

On the other hand, at the higher pH values tested (5.7, and 5.9), the final butyric acid concentrations were both lower; 26.3 g/L and 16.6 g/L for pHs 5.7 and 5.9 respectively while the acetic acid final concentrations were 1.0 g/L and 1.5 g/L respectively. It is thought that this may be attributed to the enhanced growth of *B.* sp. SGP1 at these higher pH values, leading to competition for the sugars produced. On the other hand, the final butyrate and acetic acid concentrations with the lowest pH tested, *i.e.*, 5.3 were 20.8 and 10.0 g/L respectively. It was also observed that the glucose concentration was lower than 0.1 g/L throughout the course of fermentation when the pH was 5.5, 5.7, and 5.9 while, at pH 5.3, it was increasing constantly during the course of fermentation to reach 10.0 g/L near the end of the fermentation (Data not shown). It is also worthy to note that, with an optimum pH of 5.5, the sucrose hydrolysis was higher during the early stages of the fermentation than during the latter (>100 h), suggesting that the *Bacillus* strain was limited in its growth or activity.

### The ability of the co-culture to grow in RCM medium without previous purging with an inert gas

**Figure 8:**
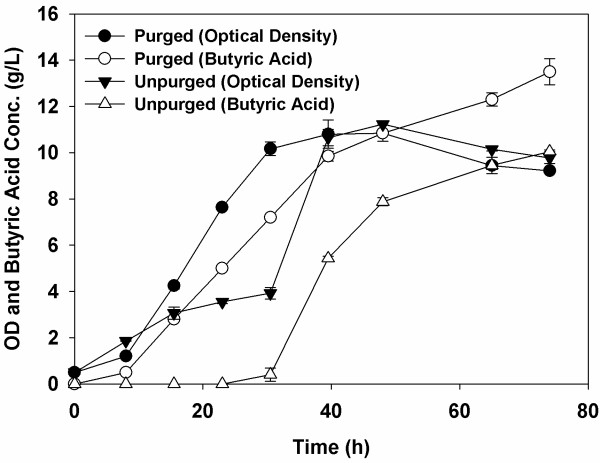
**Growth of and butyric acid production by *B. *sp. SGP1 and *C. tyrobutyricum *ATCC 25755**^**T **^**co-cultures with and without purging of the medium with argon gas.** The cultures were grown in serum bottles at 37°C using RCM supplemented with sucrose.

*C. tyrobutyricum* ATCC 25755^T^ is a strict anaerobe and sensitive to oxygen so the culturing medium needs to be purged with an inert gas like nitrogen or argon prior to inoculation to provide anoxic conditions. However, this purging contributes to the total cost for butyric acid production. Since *B.* sp. SGP1 strain is a facultative anaerobe, we wanted to test the ability of this co-culture to grow in RCM medium without prior purging. We found that the co-culture could grow successfully and produce butyric acid, albeit after a longer lag time (Figure 8). It appears that *B.* sp. SGP1 grew and consumed the oxygen present in the medium, thus providing the anoxic conditions needed for the growth of *C. tyrobutyricum* ATCC 25755^T^. This finding is important since it suggests that fermentations using these two bacteria can be performed using media without purging, allowing industries to reduce their production costs.

## Conclusions

Although *C. tyrobutyricum* ATCC 25755^T^ alone is unable to utilize sucrose, using a co-culture of *C. tyrobutyricum* ATCC 25755^T^ and *B.* sp. SGP1*,* this study demonstrates that sucrose can be utilized as a carbon source for butyric acid production. In addition, this co-culture offers also the benefit of a greater selectivity for butyric acid. Another advantage of this co-culture is its ability to produce butyric acid at a relatively high yield, which is similar to the yield obtained by pure cultures of *C. tyrobutyricum* ATCC 25755^T^ grown on glucose. One drawback for this proposed co-culture is the low productivity during fed-batch fermentations. This can be improved in future studies by changing the medium composition or the fermentation mode to optimize the growth kinetics of the two strains. Likewise, future work may also include using this novel *B.* sp. SGP1 strain or other *Bacillus* strains in similar co-cultures with *C. tyrobutyricum* ATCC 25755^T^ for production of butyric acid but from other carbon sources, such as starch or cellulose.

## Materials and methods

### Reagents

All of the chemicals and reagents used were purchased from the Sigma-Aldrich Co. USA, unless mentioned otherwise. The reagents, when available, were molecular biology grade. All solutions were prepared using these reagents and sterile distilled water.

### Bacterial strains and culturing methods

*B.* sp. SGP1 was isolated from a waste water sludge sample obtained from the Hyum Ki So Hwa Jo anaerobic digestion tank, Seoul, South Korea. This strain was co-cultured with *C. tyrobutyricum* ATCC 25755^T^ in reinforced clostridial media (RCM). The media was made fresh using the individual components and supplemented with 40.0 g/L of either sucrose for the co-culture tests or *B.* sp. SGP1 monocultures or glucose for *C. tyrobutyricum* ATCC 25755^T^ monocultures. The composition of the RCM medium was as follows: beef extract (Difco, USA) 10.0 g/L, peptone (Difco, USA) 5.0 g/L, yeast extract (Difco, USA) 3.0 g/L, tryptone (Difco, USA) 5.0 g/L, starch 1.0 g/L, sodium acetate 3.0 g/L, and NaCl 5.0 g/L. The medium was purged with argon gas and aliquoted into 60 ml serum bottles (20 ml in each), after which it was sterilized by autoclaving at 121°C for 20 minutes. For some experiments, P2 medium was used instead of RCM. The composition of the P2 medium was as follows: yeast extract (Difco, USA) 1.0 g/L, K_2_HPO_4_ 0.5 g/L, KH_2_PO_4_ 0.5 g/L, ammonium acetate 2.2 g/L, p-aminobenzoate 0.001 g/L, thiamin 0.001 g/L, biotin 0.00001 g/L, MgSO_4_.7H_2_O 0.2 g/L, MnSO_4_.H_2_O 0.01 g/L, FeSO_4_.7H_2_O 0.01 g/L, and NaCl 0.01 g/L. Unless else is mentioned, incubation and growth of the cultures was done in a shaking incubator at 37°C and 200 rpm.

### Analytical methods

Quantitative analysis of the volatile fatty acids (acetic and butyric acids) was done using GC (gas chromatography) (6890N, Agilent Technology, USA). Helium was used as the carrier gas. The oven temperature was programmed to increase from 50 to 240°C at a rate of 10°C/min. The temperature of the injection port and FID (Flame Ionization Detector) were 250°C. The concentration of the acids was determined according to a standard calibration curve. For analysis of the sugars and lactic acid, an HPLC (1200 Series, Agilent Technology, USA) with an RID (Refractive Index Detector) was used under the following conditions: mobile phase, 0.01M sulfuric acid; column, Aminex HPX-87H (dimensions - 300*7.8, Biorad, USA); flow rate, 0.5 ml/min; temperature, 40°C. The optical densities of the samples were measured using spectrophotometer (UV-mini 1240, Shimadzu, Japan) at 600 nm. To prove that [1, 2, 3 ^13^C_3_] lactic acid is converted to butyric acid and not acetic acid by *C. tyrobutyricum* ATCC 25755^T^, spent culture broth was analyzed using GC equipped with a Pegasus 2D TOF-MS (LECO, St. Joseph, MI, USA) for detecting the presence of ^13^C in acetic and butyric acids. The column was HP-INNOWAX Polyethylene glycol (PEG) capillary column (17 m × 0.32 mm internal diameter, 0.25 μm film thickness, Agilent Technology, USA). The oven temperature was programmed to increase from 50°C to 150°C at a rate of 10°C/min. Helium was used as the carrier gas with a flow rate of 3 ml/min. The ionization mode employed was EI (electron impact ionization) with a scan rate of 100 spectra/sec and mass range of 40~500 m/z. The ion source temperature was 230°C [24].

### Identification of the isolated *Bacillus* strain

The 16S rRNA gene sequence of the strain SGP1 was used as a query against the EzTaxon-e database [25,26] and the 16S rRNA gene sequence of relatives were retrieved. The construction and evaluation of the phylogenetic trees were performed as described previously [27]. The alignment and phylogenetic analysis were performed using MEGA5 [28]. The 16s rRNA gene sequence of the strain SGP1 was submitted to GenBank (http://www.ncbi.nlm.nih.gov) and is registered under accession number HQ188291.1.

### Fermentation conditions

Fed-batch fermentations were done using a 3 L capacity bioreactor (Fermentec Co. Ltd., Korea) with an initial working volume of 1 L. The medium was RCM supplemented with 60.0 g/L sucrose (Sigma-Aldrich Co. USA). After autoclaving, the medium was purged with filtered oxygen-free argon gas. Overnight pre-cultures of the two strains were inoculated into the reactor at a concentration of 5% (v/v) for each strain. All fermentations were performed using agitation (200 rpm) and the pH was adjusted to the required values (5.3 – 5.9) by addition of a 10% ammonia solution, prepared by diluting an ammonium hydroxide solution (Cat # 320145, Sigma-Aldrich Co. USA) using sterile distilled water.

### Observing levansucrase enzyme on an SDS-PAGE gel

A one-day (24 h) aerobic culture of *B.* sp. SGP1 was centrifuged and the supernatant was taken as a source for levansucrase enzyme. Concentration of the supernatant proteins was done through addition of acetone, centrifugation and then re-dissolving the precipitated proteins in phosphate buffer (pH 7.0). SDS-PAGE gel was then carried out as described by Laemmli with a 7% (w/v) acrylamide gel. After migration, the gel was cut into two halves. One half (containing the protein markers) was stained with Coomassie blue. The other half (containing the samples) was stained using periodic acid-Schiff reagent method [29-32] as follows: first, the gel was washed in a washing buffer containing 10 mM phosphate buffer (pH 7), CaCl_2_ (2 mM), and Tween 80 (10.0 g/L). Then the gel was incubated in the same solution but with 50 g/L sucrose, for 24 h at 37°C. After that, the formed levan was fixed on the gel through immersing in 70% ethanol, then the oxidizing solution (periodic acid o.7% (w/v), acetic acid 5% (w/v)) was added, followed by sodium bisulfate washing, and finally immersing in Schiff’s reagent (Sigma Aldrich, USA, Cat #; 3952016).

### Sucrase and levan forming activity assay

The sucrase and levan forming activities at different temperatures and different pH values were determined by adding 50 μl of spent media, *i.e.* without cells, from a 20 h old *B.* sp. SGP1 culture to 950 μl of the reaction mixture (0.5 M sucrose solution in phosphate buffer) [18]. To study the effect of the temperature, the pH was first adjusted to 6.6 before incubation at the test temperatures. Likewise, a temperature of 37°C was used when studying the effect of the pH. The incubation was performed for 10 h, after which, the sucrase and the levan forming activities were measured. Sucrase activity was determined by measuring the glucose liberated using glucose kits (Merck, Germany, Cat # 1.16720.0001), while levan formed was measured using the following procedure: 500 μl of the sample was added to 750 μl ethanol (to precipitate levan), and then the mixture was centrifuged. The precipitate was then washed with ethanol again, dried and then boiled with 0.1 N HCl for 30 min. Finally, the liberated fructose was analyzed using HPLC [14,18].

## Abbreviations

RCM: Reinforced clostridial medium; GC: Gas chromatography; HPLC: High-performance liquid chromatography; FID: Flame ionization detector; RID: Refractive index detector; GC-MS: Gas chromatography–mass spectrometry; EI: Electron impact ionization

## Competing interests

The authors declare no competing interests.

## Authors’ contributions

MD performed the experiments, analyzed the data and drafted the manuscript; SK and BSJ helped in some experimental work. YU and RJM contributed in data interpretation; MD and RJM wrote and revised the manuscript; MD and BIS designed the study; BIS coordinated the study. All authors read and approved the final manuscript.

## Supplementary Material

Additional file 1: Figure S1 Neighbor-joining tree based on nearly complete 16S rRNA gene sequences showing the relationships between the strain SGP1 and its related strains. The percentage numbers at the nodes are the levels of bootstrap support based on neighbor-joining analyses of 1000 resampled data sets. *Marinococcus halophilus* DSM 20408^T^ (X90835) was used as the out group (not shown). Scale bar: 0.01 nucleotide substitution per position. Click here for file

Additional file 2: Figure S2 Effect of temperature on sucrase activity of the supernatant of *B.* sp. SGP1. *B.* sp. SGP1 was grown for 22 h in RCM supplemented with sucrose, then 50 ul of the supernatant was taken and added to 950 μl of sucrose solution (0.5 M in phosphate buffer, at pH 6.6, and different temperature) and then the liberated glucose was measured as described in the Materials and Methods. Click here for file

Additional file 3: Figure S3 Growth of *B.* sp. SGP1 in RCM adjusted to different pH values. This experiment was done in serum bottles without previous purging with argon and the OD_600_ was measured after 6 h of growth at 37°C in a shaking incubator. Click here for file

Additional file 4: Figure S4 Effect of temperature on butyric acid production by the co-culture. This experiment was done in serum bottles using RCM supplemented with sucrose. Click here for file
