# Supplementary figures and images for: Co-culturing a novel Bacillus strain with Clostridium tyrobutyricum ATCC 25755 to produce butyric acid from sucrose
Source: Biotechnol Biofuels. 2013 Mar 4;6:35. doi: 10.1186/1754-6834-6-35 (PMC3610116; doi:10.1186/1754-6834-6-35)

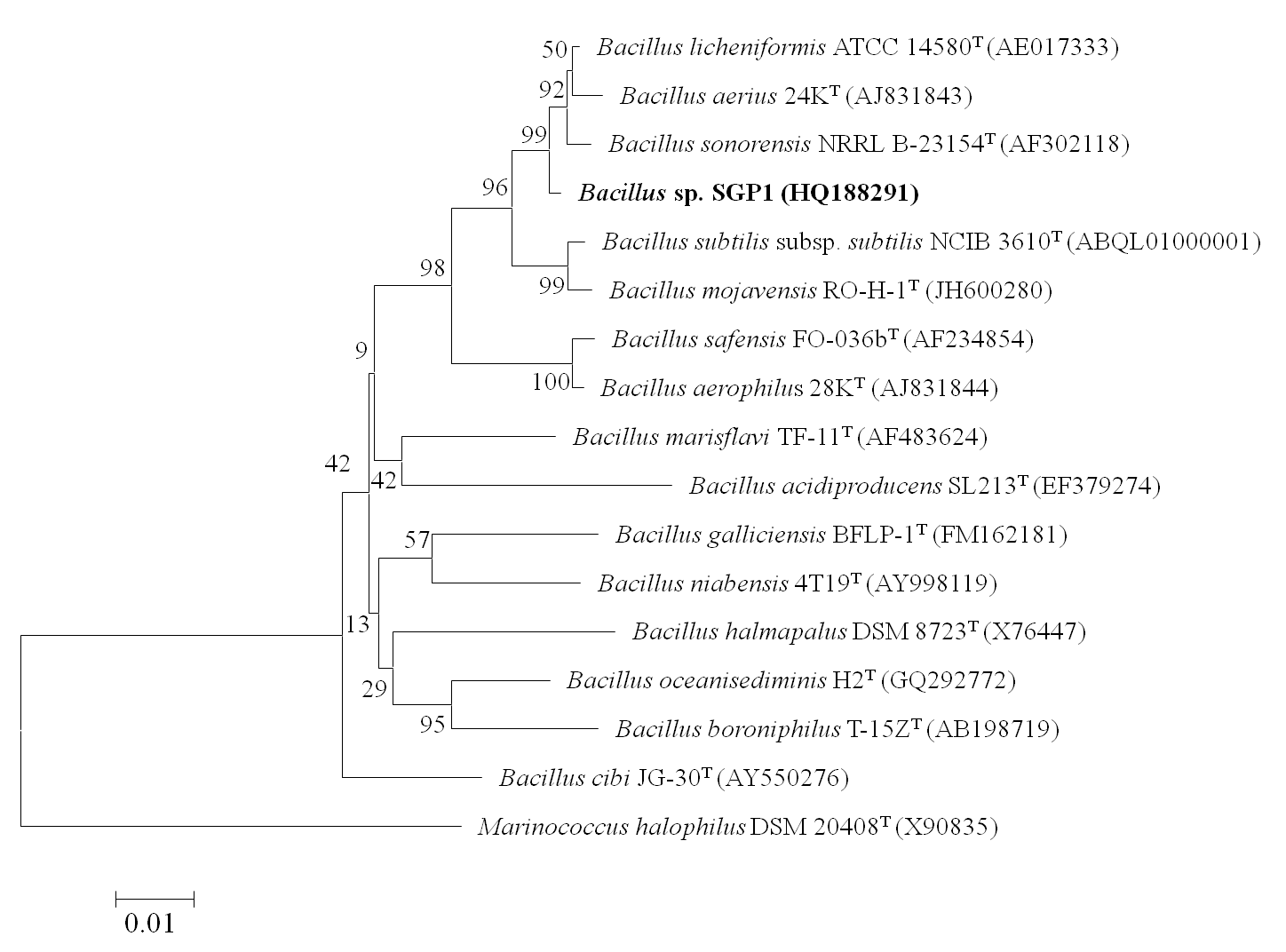

Supplement: Additional file 1: Figure S1 — Neighbor-joining tree based on nearly complete 16S rRNA gene sequences showing the relationships between the strain SGP1 and its related strains. The percentage numbers at the nodes are the levels of bootstrap support based on neighbor-joining analyses of 1000 resampled data sets. Marinococcus halophilus DSM 20408T (X90835) was used as the out group (not shown). Scale bar: 0.01 nucleotide substitution per position. [file 1754-6834-6-35-S1.tiff]
